# Why older workers work beyond the retirement age: a qualitative study

**DOI:** 10.1186/s12889-017-4675-z

**Published:** 2017-08-22

**Authors:** Ranu Sewdas, Astrid de Wind, Lennart G.L. van der Zwaan, Wieke E. van der Borg, Romy Steenbeek, Allard J. van der Beek, Cécile R.L. Boot

**Affiliations:** 1 Department of Public and Occupational Health, Amsterdam Public Health research institute, VU University Medical Center Amsterdam, Van der Boechorststraat 7, 1081 BT Amsterdam, The Netherlands; 2 Body@Work, Research Center on Physical Activity, Work and Health, TNO-VU/VUmc, Amsterdam, The Netherlands; 3 Netherlands Organization for Applied Scientific Research, TNO, Department of Work, Health & Care, Schipholweg 77-89, 2316 ZL Leiden, The Netherlands; 4 Department of Medical Humanities, Amsterdam Public Health research institute, VU University Medical Center Amsterdam, De Boelenlaan 1089a, 1081 HV Amsterdam, The Netherlands

**Keywords:** Ageing, Bridge employment, Employment participation, Retirement, Qualitative research

## Abstract

**Background:**

The aims of the present study were to: 1) gain insight into reasons for working beyond the statutory retirement age from older workers’ perspectives, and 2) explore how the domains of the research framework Study on Transitions in Employment, Ability and Motivation (STREAM) can be applied to working beyond retirement age.

**Methods:**

A qualitative research design included individual interviews (*n* = 15) and three focus groups (*n* = 18 participants) conducted with older workers aged 65 years and older continuing in a paid job or self-employment. Interview participants were recruited from an existing STREAM cohort study. Focus group participants were recruited from companies and employment agencies. The data were subjected to thematic analysis.

**Results:**

The most important motives for working beyond retirement age were maintaining daily routines and financial benefit. Good health and flexible work arrangements were mentioned as important preconditions. The themes emerging from the categorization of the motives and preconditions corresponded to the domains of health, work characteristics, skills and knowledge, and social and financial factors from the STREAM research framework. However, our analysis revealed one additional theme—purpose in life.

**Conclusion:**

This study offers important new insights into the various preconditions and motives that influence working beyond retirement age. In addition, the five domains of the STREAM research framework, including the additional domain of ‘purpose in life’, seem to be applicable to working beyond retirement age. This knowledge contributes to the development of work-related interventions that enhance older workers’ motivation to prolong their working lives.

## Background

In the Netherlands and many other Western countries, the population is ageing rapidly due to lower fertility rates, longer life expectancies and maturing *baby boomers* [1]. In fact, the proportion of persons 65 years or above in the Netherlands is estimated to reach a peak of 25% in 2040 [2]. By comparison, in 2010 this percentage was 15% [2]. To reduce the effects of an ageing society on social security systems, the Dutch government has been implementing reforms to encourage older workers to prolong their working lives. This is reflected in the increase of the statutory retirement age for people who have lived or worked in the Netherlands from 15 to 65 years and are eligible to receive an old-age government pension. The benefit level depends on the retiree’s domestic situation, and guarantees 70% of the worker’s net minimum wage. The statutory retirement age was raised from 65 years in 2012 to 67 years in 2021 [3]. Thus, the average Dutch retirement age increased from 61 years in 2006 to 64.4 years in 2015 [4]. It is relevant to understand older workers’ motivations for prolonging their work participation past retirement in light of the policy focus on enhancing prolonged working lives.

Prolonged work participation is already visible in older workers who decide to continue their engagement in work activities beyond the statutory retirement age. This phenomenon of working beyond retirement age is also called ‘bridge employment’ and refers to having paid work after receiving an old age pension and spans the period from full-time work to full retirement. In the United States, employees aged 65 years and older often participate in paid employment after retirement [5, 6]. In this age group, the labour force participation rate increased from 12.1% in 1990 to 16.1% in 2010 [7]. This trend is also becoming more common in some of the other Organization for Economic Co-operation and Development (OECD) countries. In the Netherlands, for example, the net labour participation rate for the 65–75 age group has doubled from 5.5% in 2003 to 11.0% in 2014 [8].

In recent years, an increasing amount of literature has been published on factors associated with working beyond the statutory retirement age [9–12]. De Wind et al. [9] found that work motivation, health and financial situations all influenced working beyond retirement. Examples of work-related factors associated with bridge employment are the extent to which people enjoy their work (i.e. job flexibility in working hours or less demanding jobs) and find it fulfilling [11]. Moreover, it was shown that social factors, such as having a working spouse and children to support, were positively associated with the desire to engage in bridge employment [12].

To date, only a few studies have used a qualitative research design to explore the reasons that older workers extend their working life while receiving a pension. For example, Reynolds et al. [13] identified three themes as important benefits for working beyond the age of 65: increasing financial security, maintaining health, and continuing personal development. Furthermore, several theoretical perspectives provide the opportunity to gain a better understanding of the decision to prolong work participation. For example, Atchley’s [14] Continuity Theory suggests that older individuals are more likely to maintain similar routines, structures and familiar social networks to that of their earlier years.

From the aforementioned studies and theoretical perspective, it can be concluded that the decision to prolong work participation is not driven by a single factor, but rather should be considered as multifactorial. However, to date there is no available theoretical model or framework that includes an overview of all factors that explain why older workers prolong their work participation beyond retirement age. Recently, based on the literature, the Study on Transitions in Employment, Ability and Motivation (STREAM) research framework proposed to capture the complexity of determinants that influence work productivity and employment transitions [15]. According to this framework, transitions in employment status are influenced by determinants in five domains: health, job characteristics, skills and knowledge, social factors and financial factors. Since a theoretical model or framework for working beyond retirement age is lacking, it is important to explore if and how the domains of the STREAM research framework can be applied to this phenomenon.

The aims of the present study were to: 1) gain insight into the reasons for working beyond the statutory retirement age from the perspectives of older workers (65 years and above), who are continuing in a paid job or self-employment, and 2) explore how the STREAM research framework’s domains can be applied to working beyond retirement age.

## Methods

### Design

A qualitative research design was used including individual semi-structured telephone interviews and focus groups among older employees and self-employed persons aged 65 years or above between February and June 2016. The interviews collected a first inventory of themes explaining reasons why older workers work beyond retirement age. The focus groups were conducted to validate the themes that emerged from the individual interviews and to obtain more in depth information about how the themes related to working beyond retirement. The consolidated criteria for reporting qualitative research (COREQ) were taken into account by the research team [16]. A team of academic researchers conducted the study; RS, AW, and WvdB have participated in qualitative research training and were experienced in conducting qualitative studies.

### Participant selection and recruitment

Interview participants were recruited from an existing STREAM prospective cohort study. STREAM’s aim is to identify the circumstances in which persons aged 45 to 64 years prolong their working life while maintaining good health and good work productivity. Detailed information on STREAM can be found elsewhere [15]. Participants from STREAM aged 65 years or above, who had participated in the fifth wave of data collection in 2015, reported having a post-retirement paid job or to be self-employed, and had given permission to be contacted for additional research were eligible for participation. To ensure heterogeneity, participants were purposefully selected based on educational level, gender, and health status. This is also known as maximum variation sampling [17]. We selected participants by educational level, since differences in reasons for working beyond retirement might exist due to specific work exposures (e.g. physical working conditions). In addition, multiple reasons might apply for working beyond retirement for both men and women and those with poor or good health. Between January and February 2016, participants were contacted by telephone; the purpose of this study was explained and their consent was documented. The sampling ended when no new information arose during the interviews thus implying that data saturation had been achieved [18].

For the focus group participants three recruitment locations were used to identify persons aged 65 years or above: employment agencies, a university and hospital. Since the purpose was to validate the results of the individual interviews, we conducted focus groups with another population. Participants were purposefully selected based on the same criteria as for the interviews, that is gender, educational level, and health status. Between March and May 2016, the participants were approached either by phone or invitation letter detailing the purpose of the study. Participants of the focus groups were offered transport expenses and a gift card for 15 euro. Sampling for the focus groups stopped when data saturation was reached.

### Data collection and data analyses

#### Semi-structured telephone interviews

In the first part of the study, 15 semi-structured telephone interviews were conducted by a female and a male researcher, RS and LvdZ. A semi-structured interview guide on the following topics was created: 1) reasons for working beyond the retirement age, 2) considerations about leaving work, 3) the timing at which people decide to remain active or retire, 4) persons who played a role in their decision, and 5) planning for the future (e.g. retirement). Before starting the interview, the interviewer introduced him/herself, and informed the participant about the purpose of this study, anonymity and confidentiality. The interviews lasted between 30 and 60 min. During all interviews, the interview-guide form on the computer was used to take detailed notes about the participants‘ responses. The telephone interviews were not audio recorded. After three interviews, the interview guide topics and detailed notes were evaluated by RS and LvdZ. The evaluation did not lead to adjustments of the interview guide, however, both interviewers agreed on the structure for each interview’s notes.

Data analysis was an ongoing, iterative process, including the continuous comparison of new data with previous data to establish emerging themes. Individual interviews were analysed for themes in three steps [19]. First, the notes from three individual interviews were manually open coded. Researcher triangulation was used for coding: two researchers, RS and LvdZ, independently coded the interview notes [20]. The aim of this step was to understand why persons who were interviewed were working beyond the retirement age. Next, the codes and coding trees were extensively discussed by RS and LvdZ, and consensus was reached. In the second step, the remaining 12 interviews were open coded by RS and LvdZ and codes were compared by the same. In addition, data saturation was monitored. No new information arose in the last interviews. In the third step, the codes of all interviews were organized into themes by RS. The categorization of codes was extensively discussed among all authors in group meetings until consensus was achieved.

#### Focus groups

In the second part of the study, three focus groups (a total of 18 participants) were conducted. Focus groups can be used to explicate, explain or verify data [21]. The focus groups were led by the first author, RS. In the first focus group, a second female moderator, WvdB, was present. The key questions during the focus groups were: 1) what are the reasons for working beyond the retirement age, 2) what do you need to prolong your work participation, and 3) what would be reasons for you to stop working. All focus groups were held in a meeting room and lasted approximately two hours. During all focus groups, notes were taken by the assistant moderator and after written consent the focus groups were recorded on a digital voice recorder. A summary of each focus group was created and sent to all participants for verification. Participants were asked to specifically check if relevant information was missing, or whether the researchers’ interpretations corresponded to their perspectives (i.e. member check) [16]. All participants agreed with the summaries.

**Fig. 1:**
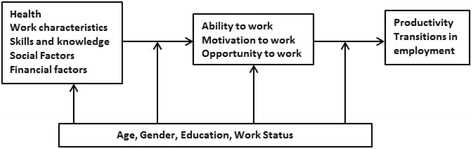
Research framework of STREAM including the five domains: health, work characteristics, skills and knowledge, social factors, and financial factors [15]

The analyses of the focus group data were conducted in five phases. In the first phase, the focus group audio tapes were transcribed verbatim. Second, all transcripts from the first and second focus groups were read line by line, and independently open coded manually by RS and AW. Next, the codes were extensively discussed by RS and AW, and consensus was reached. Third, the last focus group was open coded by RS and AW and the codes were compared by the same. Since the last focus group revealed no new reasons for working beyond retirement, data saturation was achieved. In the fourth step, all interview codes were organized into themes by RS. Code categorization from the individual interviews and focus groups were extensively discussed among all authors in group meetings until consensus on the themes were reached. Last, the five domains of the STREAM research framework were compared to the themes emerging from the analyses to explore similarities and differences for working beyond the retirement age (Fig. 1).

## Results

Table 1 presents the characteristics of the participants. The participants’ occupations varied from nurses, professors, office workers to exam supervisors.

**Table 1:** Characteristics of the participants from the individual interviews, focus groups and total participants

		Individual interviews (*n* = 15)	Focus groups (*n* = 18)	Total participants (*n* = 33)
Age (years)	Range	65–77	65–78	65–78
Gender	Males	7	15	22
	Females	8	3	11
Educational level	Low/medium	8	5	13
	High	7	13	20
Home situation	Alone	6	6	12
	Living with partner	9	12	21
Self-perceived health status	Poor	2	0	2
	Good	13	18	31
Working part-time or full-time	Part-time	13	18	31
	Full-time	2	0	2
Employment status	Employed	7	16	23
	Self-employed	8	2	10

The semi-structured telephone interviews and focus groups provided insight into the reasons for prolonging work participation beyond retirement age. Participants mentioned several reasons (divided into themes), and a number of preconditions and motives for working beyond retirement age. The research team defined a precondition as a condition that must be present before something else can occur, and a motive as something that contributes to the reason a person acts in a certain way. The themes emerging from the analysis corresponded to the five domains (i.e. health, work characteristics, skills and knowledge, social factors, and financial factors) of the STREAM research framework. In addition, one additional domain emerged from the analysis—purpose in life. An overview of all domains including citations is presented in Table 2.

**Table 2:** Overview of themes, citations, and characteristics of the participant

Theme	Citation	Participant
Health	‘*I want work, but there are also some things you have to consider of course, for example your health. That is a certain precondition.’*	Male, 70 years, employee, highly educated, focus group
	‘*Working is healthy; it keeps you young, fit and healthy. If you are working you have to stay active and use your brains.’*	Male, 68 years, employee, highly educated, focus group
Work characteristics	*‘When my supervisor offered me a contract, I asked if I could work part-time. Now I can spend more time with my family.’*	Female, 65 years, employee, highly educated, individual interview
	*‘I could not prolong my work participation. As soon as I reached the retirement age, my contract stopped and they did not want to extend my contract. They said that they did not want to hire older workers. That is why I had to stop working at that company, but currently I am able to work for a temporary employment agency.’*	Male, 69 years, employee, medium educated, focus group
	‘*Usually I have only contact with older persons. But at work I get the opportunity to be in an environment with younger persons.’*	Female, 67 years, employee, highly educated, individual interview
	*‘My partner and my clients played a role in the decision to prolong my work participation. You get a certain relationship with your clients, so you do not want to break this relationship.’*	Female, 67 years, self-employed, low-educated, individual interview
	*‘For me it is hard to say ‘no’ as a self-employed person. There is personnel shortage in the health care. They always need someone. I want to offer my help to the clients, and also to the department where I am working. They always have personnel shortage.’*	Female, 66 years, self-employed, medium educated, individual interview
Skills and knowledge	‘*Last year I had to get my certificate to drive a fork-lift truck. My supervisor, at that moment, gave me the opportunity to follow this course. I was 77 years old back then and I got a certificate. So that was quite funny actually.’*	Male, 78 years, employee, low-educated, focus group
	*‘There are only a few persons qualified for teaching this program you know. I was one of them. That is why my supervisor wanted me to continue my work participation after the pension age.’*	Male, 65 years, employee, highly educated, individual interview
Social influences	‘*It is no fun sitting at home alone. My partner has to continue working for six or seven years. But this motivation is positive for me.’*	Male, 70 years, employee, medium educated, focus group
	*‘I am living alone and I like it if can go somewhere where I can see and meet people, such as my colleagues.’*	Female, 70 years, employee, medium educated, focus group
Financial benefit	*‘The financial reason is very important for me, because I really want to keep driving in my car. I used to be an entrepreneur and it was difficult for me to save for my pension income at that time’.*	Female, 70 years, employee, medium educated, focus group
	*‘I have only one motivation to prolong my work participation and that is the money. I was not so smart as the other persons regarding saving for my pension income. Currently, I am only receiving an old age pension and I use my income as an addition to my old age pension’.*	Male, 66 years, employee, medium education, focus group
	*‘Money was not the reason to prolong my work participation. But it was something extra for me. Now I can save more money and I can spend it on travelling.’*	Male, 69 years, employee, medium educated, individual interview
Purpose in life	*‘My second reason is to continue participating in society. When you are employed, you are part of the society. You are not standing on the sideline, which is something what happens if you are ageing.’*	Male, 67 years, employee, medium educated, focus group
	‘*What I often see with peers of the same age is that after they have stopped working, they had no purpose in their life anymore and became more aware of their health problems’*	Female, 67 years, self-employed, low-educated, individual interview
	*‘Working gives you a purpose in life. If you are healthy, you can live for 20 more years and if you are going on retirement… what are you going to do? When you have 20 years left, you still want something to do in your life right? Work can give you some purpose in life in those 20 years.’*	Female, 65 years, employee, highly educated, focus group
	*‘If I am working I have to put some effort in my appearance. I love this challenge. There are some requirements when you are working. You cannot wear your sleepwear for example. Working prevents me from wearing my sleepwear the whole day.’*	Female, 65 years, self-employed, highly educated, individual interview

### Health

All participants indicated that being in good health was a necessary precondition to participate in work. As one participant said: ‘*I want to work, but there are also some things you have to consider of course, for example, your health. That is a certain precondition.’ - (Male, 70 years, employee, highly educated, focus group)* In the health domain, participating in work was the motive that offered the older worker an opportunity to stay fit and healthy. On this issue, one participant commented: ‘*Working is healthy; it keeps you young, fit and healthy. If you are working you have to stay active and use your brains.’ - (Male, 68 years, employee, highly educated, focus group)*

### Work characteristics

Most of the employed participants considered that having flexible working arrangements (working part-time, fewer obligations and working from home) was an important precondition for prolonging their work participation. Working part-time was mentioned as the most important precondition: *‘When my supervisor offered me a contract, I asked if I could work part-time. Now I can spend more time with my family.’ - (Female, 65 years, employee, highly educated, individual interview)*

For the employed participants, having an employer who allowed working beyond the retirement age was also a precondition. However, there were some negative experiences with employers regarding working beyond the retirement age: *‘I could not prolong my work participation. As soon as I reached the retirement age, my contract stopped and they did not want to extend my contract. They said that they did not want to hire older workers. That is why I had to stop working at that company, but currently I am able to work for a temporary employment agency.’ - (Male, 69 years, employee, medium educated, focus group)*

In the work characteristics domain, motives to prolong working indicated by most of the participants were: appreciation from colleagues or clients, and maintaining contact with clients or colleagues. Some participants mentioned contacts with younger colleagues as an important reason to continue working: ‘*Usually I have only contact with older persons. But at work, I get the opportunity to be in an environment with younger persons.’ - (Female, 67 years, employee, highly educated, individual interview)*

Self-employed participants expressed that contact with their clients was an important motive for remaining in the workforce after retirement age: *‘My partner and my clients played a role in the decision to prolong my work participation. You get a certain relationship with your clients, so you do not want to break this relationship.’- (Female, 67 years, self-employed, low educated, individual interview)*

Participants working in health care mentioned feeling responsible for their clients was a reason for prolonging their work participation: ‘*For me it is hard to say ‘no’ as a self-employed person. There is personnel shortage in the health care. They always need someone. I want to offer my help to the clients, and also to the department where I am working. They always have personnel shortage.’ - (Female, 66 years, self-employed, medium educated, individual interview)*

### Skills and knowledge

Motives related to skills and knowledge that participants mentioned for prolonging work past retirement included: utilization of abilities, ability to pass on skills and knowledge, and learning new skills and knowledge: ‘*Last year I had to get my certificate to drive a fork-lift truck. My supervisor, at that moment, gave me the opportunity to follow this course. I was 77 years old back then and I got a certificate. So that was quite funny actually.’ - (Male, 78 years, employee, low educated, focus group)*

One highly educated person mentioned that he was asked to prolong his work participation, because of his skills and knowledge: *‘There are only a few persons qualified for teaching this program you know. I was one of them. That is why my supervisor wanted me to continue my work participation after the pension age.’ - (Male, 65 years, employee, highly educated, individual interview)*

### Social influences

Not wanting to sit at home alone was also mentioned as a motive to continue working. Participants with a working partner commented: ‘*It is no fun sitting at home alone. My partner has to continue working for six or seven years. But this motivation is positive for me.’ - (Male, 70 years, employee, medium educated, focus group)*

Similarly, the majority of the participants without a partner stated that living alone was a reason for prolonging their work participation: *‘I am living alone and I like it if can go somewhere where I can see and meet people, such as my colleagues.’ - (Female, 70 years, employee, medium educated, focus group)*

### Financial benefit

Many participants mentioned financial benefit as a motive for working beyond retirement age. For some participants, prolonging their work participation was a financial necessity (i.e. shortfall of pension income, higher living standards, having to pay a mortgage): *‘The financial reason is very important for me, because I really want to keep driving in my car. I used to be an entrepreneur and it was difficult for me to save for my pension income at that time’. - (Female, 70 years, employee, medium educated, focus group) ‘I have only one motivation to prolong my work participation and that is the money. I was not so smart as the other persons regarding saving for my pension income. Currently, I am only receiving an old age pension and I use my income as an addition to my old age pension’. – (Male, 66 years, employee, medium education, focus group).*

Whereas financial security was not the most important reason or sole reason for the majority of the participants to prolong their work participation (especially for those who were medium or highly educated) it did represent extra income for spare-time activities, grandchildren or savings. As one participant put it: *‘Money was not the reason to prolong my work participation. But it was something extra for me. Now I can save more money and I can spend it on travelling.’ - (Male, 69 years, employee, medium educated, individual interview)*

### Purpose in life

In the domain of purpose in life, the participants indicated three specific motives for remaining in the work force. First, all participants identified their contribution and participation in society as motives to remain working past retirement age: ‘*My second reason is to continue participating in society. When you are employed, you are part of the society. You are not standing on the sideline, which is something what happens if you are ageing. - (Male, 67 years, employee, medium educated, focus group)*

Second, all participants stated that they had worries about their life as a retiree: ‘*What I often see with peers of the same age is that after they have stopped working, they had no purpose in their life anymore and became more aware of their health problems’ - (Female, 67 years, self-employed, low educated, individual interview)* As another participant said: *‘Working gives you a purpose in life. If you are healthy, you can live for 20 more years and if you are going on retirement… what are you going to do? When you have 20 years left, you still want something to do in your life right? Work can give you some purpose in life in those 20 years.’ - (Female, 65 years, employee, highly educated, focus group)*

Third, one of the most mentioned and important motives to continue working was that work provided a practical purpose in life, namely the opportunity to maintain daily routines: *‘If I am working I have to put some effort in my appearance. I love this challenge. There are some requirements when you are working. You cannot wear your sleepwear for example. Working prevents me from wearing my sleepwear the whole day.’ - (Female, 65 years, self-employed, highly educated, individual interview)*

## Discussion

Older workers participating in work beyond the statutory retirement age indicated several preconditions and motives for staying in the work force. According to our results, the domains of health, work characteristics, skills and knowledge, social factors, and financial factors derived from the STREAM research framework can be applied to working beyond retirement age. In addition, motives were identified in one additional domain—purpose in life.

Being in good health was mentioned as an important precondition for working beyond retirement age in our study. This result is consistent with findings from Dingemans et al. [22], who found that healthy men and women are more likely to participate in bridge employment. In line with this, the opposite appears to be the case for early retirement since previous studies have shown that poor health influences early retirement [23]. Nevertheless, it has also been suggested that older healthy workers decide to retire early to enjoy their life or fulfil other goals [23, 24]. A possible explanation for this might be that there is an interplay between being in good health and other factors in a person’s decision to work beyond retirement age. It is important to note that our results show that staying fit and healthy was a motive for prolonging work participation. These results are in agreement with Reynolds et al.’s [13] qualitative findings that subjective benefits for working beyond 65 were keeping physically active and maintaining physical as well as mental health.

With respect to the domain of work characteristics, a significant finding is that the participants prefer working part-time over working full-time. This may be explained by the fact that older workers want to have a satisfying balance between work and relaxation in their lives [13]. In another study, having high control over work time compared to low control was found to have a positive influence on working longer [25]. Furthermore, Ulrich et al. [26] showed that not only did flexibility in working hours influence bridge employment, but flexibility in choosing projects, working at one’s own pace and working in a familiar and comfortable environment did as well.

Regarding the domain of skills and knowledge, our results further support the finding from a previous study that older workers in bridge employment are satisfied with their work, because they have the ability to keep learning and demonstrate their competency [26]. In addition, a previous study demonstrated that persons who have a higher focus on skills and knowledge development are less likely to retire early [27]. Since the development of skills and knowledge are likely to be linked to educational levels and occupation, this is in line with our findings.

With regard to the role of social influences, a previous qualitative study on factors influencing early retirement showed that having a non-working partner was a pull factor that attracted individuals towards early retirement, because they wished to do enjoyable activities with their non-working partner [28]. Our results also showed that retirement could be less attractive in cases where there was a working partner, since workers did not want to sit at home alone. It is surprising that in our study financial benefit was rarely mentioned as the sole reason for prolonging work participation. This result matches findings of an earlier qualitative study, wherein less than half of the participants explicitly mentioned additional income as a reason for prolonging work participation [13]. That said, our results did indicate that the influence of financial benefit might differ for low- versus highly educated persons since those with a low education may experience more financial difficulties [29]. Workers who are well-paid and have a good pension may choose to work beyond retirement for positive reasons (e.g., extra income for spare time activities, grandchildren or savings), while those in financial difficulty may be forced to work beyond retirement which might be unsatisfying and physically demanding. This situation may further increase existing social inequalities.

The five domains of the STREAM research framework correspond largely to the themes that emerged from our data suggesting that this framework can be applied to working beyond retirement age. Additionally, our results indicate that the domains in the STREAM research framework could be complemented by one extra domain—purpose in life. Furthermore, the motives found in the domain of purpose in life can be related to Atchley’s Continuity Theory, which underlines the importance of structure in older adults’ lives [14]. This was reflected in our results since one of the most mentioned motives was the importance of maintaining daily routines, thus implying that bridge employment is an efficient way to maintain daily structures.

Our study has several strengths and limitations that should be taken into account when interpreting the results. A strength of this study is the qualitative design, which allowed us to explore insights and motives. We were able to explicate and validate the individual interview data with focus group data. Another strength in this study was capturing an overview of the factors involved in working beyond the retirement age in a theoretical framework. We had a heterogeneous sample with regard to educational level. Unfortunately, we only managed to include a limited number of females and participants with less than good health, which was a limitation. Nevertheless, this might be partly explained by the fact that males and healthy persons are more likely to participate in bridge employment and being healthy was a precondition for prolonging work participation in our study [10, 22]. The ‘healthy worker effect’ may explain the majority of the unhealthy workers departing the workforce with a disability benefit, thus leaving a selection of healthy workers available for working beyond retirement age [30]. Another limitation was that the analyses of the telephone interviews were based on interviewer notes. That said, since the telephone interviews were semi-structured, we do not believe that this approach adversely influenced our results. Moreover, the focus groups followed findings from the telephone interviews, and as a result allowed for soliciting information we might have missed during the telephone interview phase.

The findings of this study have a number of practical implications and give direction to future research. First, this study confirms that the decision to work beyond retirement is driven by multiple factors. Accordingly, policies and interventions to stimulate prolonged working lives should focus not only on the financial incentives, but also on the diversity of other factors. Future research is needed to determine how all dimensions (and motives) might interact for working beyond retirement. Second, according to preconditions found in the domain of work characteristics, further research is needed to explore employers’ perspectives. Since being in good health was another important precondition for remaining in the workforce, in the context of policies to increase the statutory retirement age, future research is needed to gain more insight into how recent reforms may influence work participation among the large group of older workers with less than good health.

## Conclusion

This was the first study in the Netherlands to demonstrate that various preconditions and motives influence working beyond the retirement age. The second major finding was that the domains of health, work characteristics, skills and knowledge, social factors, and financial factors from the STREAM research framework are applicable for working beyond retirement age. Furthermore, an additional domain, purpose in life, arose from the interviews. Taken together, this knowledge could contribute to the development of work-related interventions that enhance a prolonged working life in older workers, such as increased work accommodations (e.g. shorter working day, work from home, and an opportunity to learn new skills).
